# The clinical outcomes of total femur prosthesis in patients with musculoskeletal tumors

**DOI:** 10.1051/sicotj/2019020

**Published:** 2019-06-28

**Authors:** Takuya Kakimoto, Akihiko Matsumine, Kunihiro Asanuma, Takao Matsubara, Tomoki Nakamura, Akihiro Sudo

**Affiliations:** 1 Department of Orthopaedic Surgery, Mie University Graduate School of Medicine 2-174 Edobashi Tsu City Mie 514-8507 Japan; 2 Department of Orthopedics and Rehabilitation Medicine, Faculty of Medical Sciences, University of Fukui Matsuoka-shimoaizuki 23-3 Eiheiji Fukui 910-1193 Japan

**Keywords:** Total femur prosthesis, Total femur replacement, Musculoskeletal tumor, Kyocera Modular Limb Salvage System, Orthopaedic Salvage System

## Abstract

*Introduction:* Reconstruction using a total femur prosthesis (TFP) remains a challenging procedure in musculoskeletal tumor surgery. The purpose of this study was to show the clinical outcomes of total femur replacement (TFR) in our institute.

*Methods:* Nine patients underwent reconstruction with a TFP after the wide resection of malignant bone and soft-tissue tumors of the femur between January 2003 and April 2014. The mean age of the patients at the time of TFR was 47.5 years, and the mean follow-up period was 52.9 months. The histological diagnoses were as follows: bone sarcoma (*n* = 4), soft-tissue sarcoma invading the femoral bones (*n* = 4), and metastatic bone tumor (*n* = 1).

*Results*: The oncological outcomes were as follows: three patients achieved continuous disease free, two patients were alive with disease, and four patients died from disease. The 3- and 5-year overall survival rates were 88.9% and 55.6%, respectively. The rate of the overall survival in patients with primary bone tumors (100% at 5 years) was significantly better than that in patients with primary soft tissue sarcomas (0% at 5 years) (*p* = 0.015). A deep infection occurred postoperatively in one patient, but the patient was successfully treated with surgical debridement and revision surgery. There were no patients who showed dislocation or aseptic loosening. The mean Musculo-Skeletal Tumor Society functional score was 58.5% (46.7–80.0), with scores of 65.5% in patients with a primary bone tumor and 50.8% in those with a primary soft-tissue sarcoma.

*Discussion*: In the present study, the patients who underwent TFR due to bone invasion by soft tissue sarcoma had a worse prognosis than the bone sarcoma patients.

## Introduction

With the survival of primary or metastatic bone malignancies being improved, thanks to the advent of new chemotherapy regimens, the development of limb salvage surgical procedures has flourished [1]. However, surgery of tumors involving more than two-thirds of the femoral bone remains challenging when limb preservation is considered. These tumors are usually large with longitudinal extension, and their resection requires the removal of the whole femoral bone with extensive resection of the muscles that play an important role in the hip and knee function.

The options for treating such patients include disarticulation at the hip joint and limb-preserving surgery. Although disarticulation may be a promising procedure from the perspective of local control, the patient satisfaction can be poor due to the resultant poor limb function. Thus, limb-preserving surgery is understandably preferred by patients. However, there is no standard surgical procedure for reconstructing the defect after resection of the total femoral bone.

The available surgical procedures for reconstructing the total femoral defect include total femur replacement (TFR), allogenic bone graft with/without combination of the prosthesis [1, 2], and rotationplasty [3]. In Japan, it is difficult to obtain an allogenic bone graft fitting a large bone defect. Rotationplasty usually promises a good functional outcome, but most Japanese patients hesitate to undergo this surgical procedure for cosmetic reasons. Therefore, we reconstruct total femoral bone defects using TFR at our institution.

Only a few reports have described the clinical outcomes of TFR after resection of the total femoral bone [1, 4–6]. The purpose of this study was to show the oncologic outcomes, complications, and functional outcomes of TFR performed at our institute.

## Patients and methods

Nine patients (male, *n* = 5; female, *n* = 4) underwent reconstruction with total femur prostheses (TFPs) after wide resection of malignant bone and soft-tissue tumors of the femur between January 2003 and April 2014. The mean age of the patients at the time of TFR was 47.5 years (range: 14–78 years), and the mean follow-up period was 52.9 months (range: 9–93 months). The histological diagnoses were as follows: bone sarcoma (*n* = 4 [osteosarcoma, *n* = 3; Ewing sarcoma, *n* = 1]), soft-tissue sarcoma invading the femoral bones (*n* = 4 [undifferentiated pleomorphic sarcoma, *n* = 3; myxofibrosarcoma, *n* = 1]), and metastatic bone tumor from breast cancer (*n* = 1).

**Figure 1:**
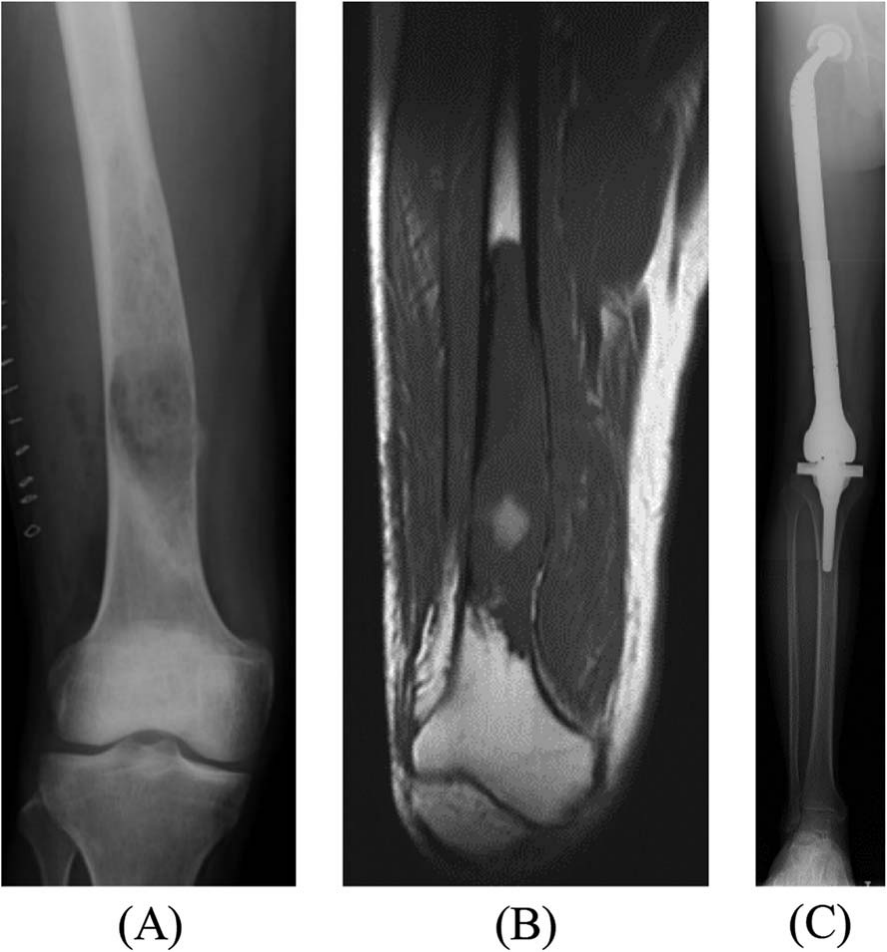
(A) X-ray of the right distal femur of 33-year-old man with a low-grade osteosarcoma. (B) Preoperative T1-weighted MRI showing the involvement of osteosarcoma in the distal two-third of the femur. (C) Radiograph demonstrating reconstruction of total femur after TFR.

**Figure 2:**
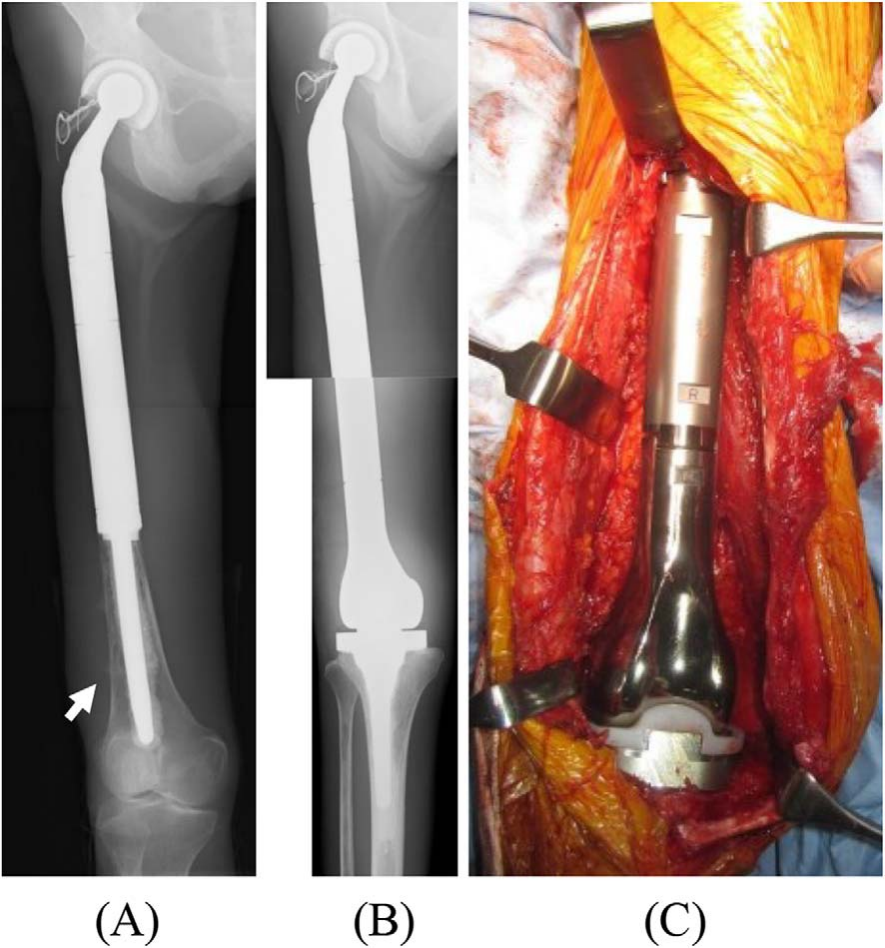
(A) X-ray of the right femur of 46-year-old woman with a local recurrence (arrow) of metastatic bone tumor from breast cancer after PFR. (B) Radiograph demonstrating the reconstruction of the right femur after TFR. (C) An intraoperative photograph after reconstruction of femoral bone using a TFR.

The TFPs were implanted at the initial surgery in three patients (Figure 1), for locally recurrent tumors in four patients (Figure 2), at the revision surgery for an aseptic loosened distal femoral prosthesis in one patient, and at the reconstructive surgery after the resection of an infected intraoperative extracorporeal irradiated bone graft in one patient.

All primary musculoskeletal tumors were staged according to the TNM classification [7]. At the time of the initial diagnosis of primary musculoskeletal tumor, four patients were stage III, three stage IIB, and one stage IB. At the time of the initial evaluation, all patients underwent a thorough oncologic examination, which included chest radiography, computed tomography, and magnetic resonance imaging (MRI). There were no distant metastases of the primary musculoskeletal tumor at the time of reconstructive surgery using TFPs. Chemotherapy was performed in six patients, and irradiation was administered in combination with the chemotherapy in five patients. Informed consent was obtained from all patients according to the guidelines of the institutional ethics review board.

Eight patients underwent TFR with the Kyocera Modular Limb Salvage System (KMLS system; KYOCERA Medical Corporation, Osaka, Japan), while one patient underwent TFR with the Orthopaedic Salvage System (Biomet Orthopedics, Inc., Tokyo, Japan). The KMLS system is a fully modular prosthetic system that was created in order to reconstruct distal femoral bone defects after tumor resection and designed specifically for Asian patients, who tend to have a relatively small anatomical architecture. The TFR of the KMLS system has a unique semi-rotating hinge knee joint that allows for a maximum flexion of 142° and an internal/external-rotation of 5° [8, 9]. The metallic parts of the KMLS system are made of light-weight and high-strength titanium alloy with good bio-compatibility and bio-stability, allowing scanning by MRI. As a result, the TFP with the KMLS system is extremely light in weight.

Cement-based or cementless fixation can be chosen for the fixation of the tibial component, depending on the surgeon’s preference. The hip joint can be reconstructed using total or bipolar hip arthroplasty. In the present series, polymethylmethacrylate cement was used for the fixation of the tibia components in four patients, while cementless fixation was performed for another four patients, and the fixation adopted in the final patient was unknown. Hip joints were reconstructed using bipolar hip arthroplasty in eight patients and total hip arthroplasty in one patient.

All surgical intervention was performed under general anesthesia by trained surgeons specialized in orthopedic oncology. All surgical resections followed the guidelines of the Japanese Orthopedic Association outlined by Enneking [10] and The JOA Committee of Tumors [11]. A wide surgical margin was obtained in seven patients. All patients received intravenous antibiotics preoperatively and postoperatively. Deep drains were used routinely, and antibiotics were given while the drains were in place. All patients were kept on bed rest and immobilized with the extremity in 30° of flexion in a bulky dressing for the first 24 h. Thereafter, the patients were started on a regimen of gentle passive range of motion (ROM) and isometric exercises, such as straight leg raisings. Full weight bearing was permitted 1 week after the surgery.

The overall survival rate was defined as the time from surgical reconstruction using TFR to the date of the final evaluation of the patients. The functional assessments were performed according to the scoring system of the Musculoskeletal Tumor Society (MSTS) [12]. The muscle strength of the knee extension was evaluated using manual muscle test (MMT) [1]. The Mann–Whitney *U* test was used to analyze the correlation between various factors and patients’ functional outcomes. The survival analysis was conducted using Kaplan–Meier curves. The survival was compared by the log-rank test. Statistical significance was determined if the two-sided value of a test was less than 0.05. Statistical analyses were performed using IBM SPSS Statistics software program, Version 22.

## Results

**Figure 3:**
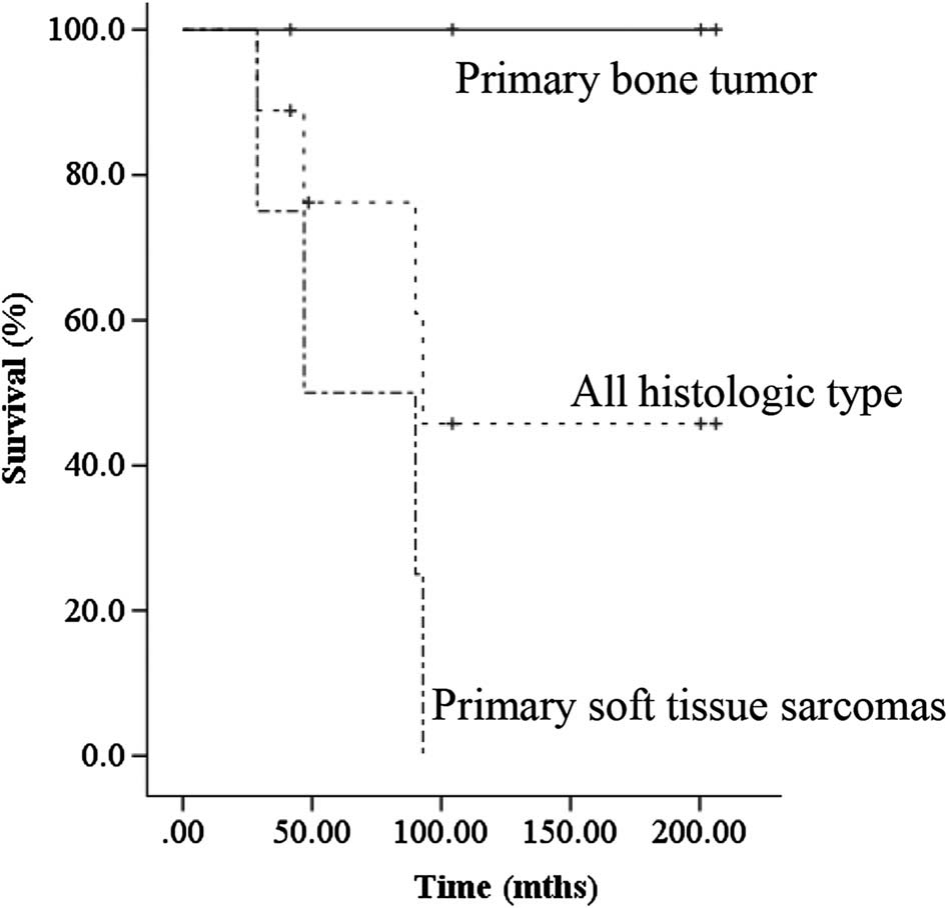
Clinical course and oncologic outcomes.

**Figure 4:**
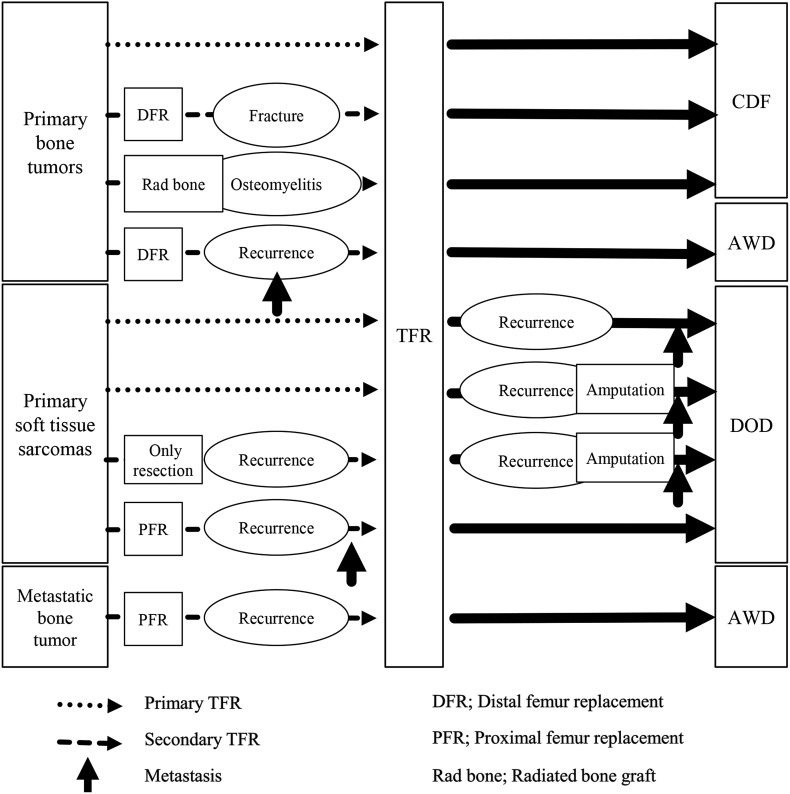
Kaplan–Meier survival curves for all patients.

The oncological outcomes were as follows: three patients achieved a continuous disease-free status, two were alive with disease (AWD), and four died of disease (Table 1). The 3- and 5-year overall survival rates (based on Kaplan–Meier estimates) were 88.9% and 55.6%, respectively. The rate of the overall survival in patients with primary bone tumors (100% at 5 years) was significantly better than that in patients with primary soft tissue sarcomas (0% at 5 years) (*p* = 0.015) (Figure 3). Three of the nine patients had local recurrence after TFR. In 2 of the 3 cases of local recurrence, the recurrence occurred after the initial resection of the primary tumors; in the other case, it occurred after resection of a recurrent tumor. Two of the three patients with local recurrence after TFR underwent hemipelvectomy. No patients had metastasis at the time of the initial treatment of the primary musculoskeletal tumor. However, five patients had metastasis after TFR, and four of them ultimately died (Figure 4).

**Table 1:** Patient characteristics.

No.	Sex	Age (years)	Diagnosis	Size (cm) (resected tumor)	Grade (resected tumor)	Adjuvant	Reason for TFR
1	M	33	OS	18 × 4 × 3	I	–	Primary tumor
2	M	27	OS	Unknown	III	Unknown	Loosening-D/F
3	M	29	Ewing’s sarcoma	Unknown	III	C	Infection-Rad bone
4	F	14	OS	6 × 4 × 3	III	C + R	LR-D/F
5	F	79	Myxofibrosarcoma	18.5 × 12 × 8	III	R	Primary tumor
6	M	58	UPS	27.5 × 14 × 10	III	C + R	Primary tumor
7	F	72	UPS	16 × 10 × 8	III	R	LR
8	M	67	UPS	6.5 × 5 × 3.5 + 4 × 3 × 2.5	III	C + R	LR-P/F
9	F	49	Metastatic bone tumor (breast)	11 × 3 × 2.5	III	C	LR-P/F
Mean		47.5					
No.	Complication	ROM		MMT	Gait	MSTS score (%)	Status (months)
		Extension (°)	Flexion (°)	Extension (°)			
1		0	50	5	Without crutches	80	CDF (104)
2		0	100	3	Without crutches	46.7	CDF (206)
3		−30	90	2	T cane	73.3	CDF (200)
4		0	45	3	Single crutch	63.3	AWD (41)
5	DVT	0	80	3	T cane	53.3	DOD (90)
6	DVT	/	/	2	Without crutches	70	DOD (46)
7		0	90	2	T cane	33.3	DOD (92)
8	Infection	0	100	2	T cane	46.7	DOD (28)
9		0	90	4	Lofstrand	60	AWD (48)
Mean		3.8	80.6	2.9		58.5	

OS, osteosarcoma; UPS, undifferentiated pleomorphic sarcoma; C, chemotherapy; R, radiotherapy; LR, local recurrence; D/F, distal femur; P/F, proximal femur; DVT, deep venous thrombosis; CDF, continuous disease-free status; AWD, alive with disease; DOD, died of disease.

Deep venous thromboses occurred postoperatively in two patients, both of whom were successfully treated with anticoagulant therapy. A deep infection occurred postoperatively in one patient; the patient was successfully treated with surgical debridement and revision surgery. There were no patients who showed dislocation or aseptic loosening.

The flexion of the knee joint ranged from 45° to 100° (mean flexion: 80.6°). The results of a manual test for knee extension muscle power ranged from grade 2 to 5 (mean: 2.8). The mean MSTS functional score was 58.5% (46.7%–80.0%). The mean MSTS functional score was 65.8% in patients with a primary bone tumor and 50.8% in patients with a primary soft-tissue sarcoma. The mean MSTS score of primary bone tumors was better than that of primary soft tissue tumors. However, no significant differences were observed. The mean MSTS functional score was 67.8% in patients with primary TFR and 53.9% in those with secondary TFR. The mean MSTS score of primary TFR was better than that of secondary TFR. However, no significant differences were observed (Table 2).

**Table 2:** Functional outcomes.

	*n*	MSTS score (%)	*p*-value
Primary bone tumors	4	65.8 (46.7–80.0)	0.2
Primary soft tissue sarcomas	4	50.8 (33.3–70.0)	
Primary TFR	3	67.8 (53.3–80.0)	0.26
Secondary TFR	6	53.9 (33.3–73.3)	
Total	9	58.5 (33.3–80.0)	

TFR, total femur replacement.

## Discussion

Limb salvage surgery has long been a standard surgical concept in musculoskeletal tumor surgery. However, when the tumor shows longitudinal extension in the femoral bone, the appropriate surgical methods remain controversial. The options for treating such patients include amputation at the proximal femoral bone, disarticulation at the hip joint, rotation plasty, and limb-preserving surgery. Although disarticulation may be a promising procedure from the perspective of local control, the patient satisfaction can be poor due to the resultant poor limb function. Thus, limb-preserving surgery is understandably preferred by patients. However, there is no standard surgical procedure for reconstructing the defect after resection of the total femoral bone.

The available surgical procedures for reconstructing the total femoral defect include TFR, allogenic bone graft with/without combination of the prosthesis, and rotationplasty [1–3]. In the present study, we showed acceptable clinical results of reconstruction using TFR after resection of the total femoral bone.

In the present study, the 3- and 5-year overall survival rates were 88.9% and 55.6%, respectively. Other reports have described 5-year overall survival rates of 32% to 44.5%, and Kalra et al. reported a survival rate of 37% at 10 years [4]. Although it is difficult to compare patients’ survivals among independent studies due to differences in patients’ characteristics, including age, gender, pathological diagnosis, tumor size, and timing of the surgery, among other factors, the survival of the patients in the present series seems similar to that described in previous reports.

The rate of the overall survival in patients with primary bone tumors (100% at 5 years) was significantly better than that in patients with primary soft tissue sarcomas (0% at 5 years) (*p* = 0.015). Indeed, all patients with primary bone tumor were alive, while all patients with primary soft tissue sarcoma were dead at the final follow-up. The prognostic factors of soft tissue sarcoma include the histological grade, tumor size, and presence of distant metastases [13]. In the present series, all patients with soft tissue sarcoma had a high grade and huge sarcoma exceeding 15 cm in diameter. We may therefore consider that the patients who underwent TFR due to bone invasion of soft tissue sarcoma may have had a worse prognosis than the bone sarcoma patients.

The complication rate after prosthetic replacement remains high. A deep infection occurred postoperatively in one patient, and one-step revision surgery was performed to avoid amputation. The rate of infection was 11.1% in our series and ranged from 3% to 22% in previous reports [1, 4–6]. Haijie et al. showed that the weighted-mean incidence of infection was 8.5% for distal femoral replacement and 16.8% for proximal tibial replacement [14]. Cannon also reported that the incidence of infection was 6.9% for proximal femoral replacement [15]. Proximal tibia replacement or TFR has always carried a higher risk of deep infection than distal femoral replacement because of its poorer soft tissue coverage. The patients in the present study had no postoperative deep infection. Adequate soft tissue coverage after reconstruction is necessary to prevent such infection. Amputation following prosthetic replacement was required in two patients (22.2%). In all cases, the amputation was due to local recurrence of disease. All of the amputations were performed in patients with recurrent high-grade soft tissue sarcomas, suggesting that soft tissue sarcoma may carry an inherently high risk of amputation. Deep venous thromboses occurred postoperatively in two cases. No previous reports described the rate of occurrence of deep venous thrombosis. TFR is an invasive procedure that requires dissection of the total femoral vein, so this surgical procedure may carry a high risk of deep venous thrombosis. A gentle surgical technique and postoperative anticoagulant therapy are necessary to prevent deep venous thrombosis after TFR.

In previous reports, the MSTS scores of the patients who received TFR ranged from 67% to 80% [1, 4–6]. In the current study, the mean MSTS score was 59%, which was worse than the values previously reported. One reason for this relatively poor functional outcome was that many secondary TFR procedures performed in patients who failed initial treatment were included in our series. Sewell et al. also reported that the function after primary TFR was better than that after secondary TFR [5]. These results suggest that the more extensive and repeated resection of muscles in secondary TFR may result in a worse limb function than with primary TFR.

## Conclusions

We described the clinical outcomes after TFR. The survival, complications, and limb function were acceptable compared with those reported in previous studies. However, the patients with soft tissue sarcoma had a worse prognosis and worse limb function than those with bone sarcoma. Therefore, the most preferable indication of reconstruction of total femoral bone defect using TFR is primary malignant bone tumor, such as osteosarcoma.
